# Acetylsalicylic acid and vorapaxar are less active, while 4-methylcatechol is more active, in type 1 diabetic patients compared to healthy controls

**DOI:** 10.1186/s12933-025-02891-6

**Published:** 2025-08-07

**Authors:** Markéta Paclíková, Lukáš Konečný, Alejandro Carazo, Kateřina Matoušová, Lenka Kujovská Krčmová, Vladimír Blaha, Alena Šmahelová, Přemysl Mladěnka

**Affiliations:** 1 Department of Internal Medicine-Metabolic Care and Gerontology, Faculty of Medicine in Hradec Králové, University Hospital, Charles University, Hradec Králové, Czech Republic; 2 Department of Pharmacology and Toxicology, Faculty of Pharmacy in Hradec Králové, Charles University, Hradec Králové, Czech Republic; 3 Department of Clinical Biochemistry and Diagnostics, University Hospital Hradec Králové, Hradec Králové, Czech Republic; 4 Department of Analytical Chemistry, Faculty of Pharmacy in Hradec Králové, Charles University, Hradec Králové, Czech Republic

**Keywords:** Diabetes mellitus, Platelets, Aggregation, 4-methylcatechol

## Abstract

**Introduction:**

It is well known that platelets from diabetic patients can be resistant to clinically used antiplatelet drugs.

**Methods:**

To assess the phenomenon in more detail, 50 adult patients suffering from type 1 diabetes mellitus (T1D) were recruited and their responses to 7 platelet aggregation inducers, as well as to 3 clinically used antiplatelet drugs (acetylsalicylic acid /ASA/, ticagrelor and vorapaxar) and one experimental compound, 4-methylcatechol, were assessed ex vivo. A control group of 50 generally healthy age-matched controls was also included for comparison.

**Results:**

T1D patients exhibited a lower aggregation reaction to 3 inducers but were conversely more resistant to the effect of ASA and vorapaxar than controls. Ticagrelor tended to be less active in T1D as well. On the other hand, 4-methylcatechol was equally or even more potent in T1D than in healthy controls. Plasma glucose levels above 7 mM were associated with lower platelet aggregation responses to four aggregation inducers. In contrast, the effect of 4-methylcatechol, unlike that of ASA, did not appear to be strongly influenced by glycemia. Further subanalyses, excluding hypertensive patients and significantly more frequently administered drugs, did not substantially modify the results.

**Conclusion:**

Conclusively, 4-methylcatechol seems to be a prototypical antiplatelet compound with a strong effect even in diabetic patients.

**Supplementary Information:**

The online version contains supplementary material available at 10.1186/s12933-025-02891-6.

## Introduction

Atherosclerosis, hyperaggregability, and high glucose levels range among the main factors for cardiovascular morbidity and mortality [1, 2]. Under normal physiological conditions, platelets remain in an inactive state and become activated solely in some circumstances, such as in overt vessel injury or the presence of advanced atherosclerosis. The first case represents an essential process needed for wound closure, while the latter leads to arterial thrombosis, the main culprit of acute myocardial infarction. The entire process of platelet activation, aggregation, and thrombus formation is highly complex, as numerous surface receptors and intricate intracellular cascades become involved [3, 4].

Type 1 diabetes mellitus (T1D) occurs due to the inability of pancreatic B-cells to produce sufficient insulin [5, 6]. Hence, it is logical that therapy for T1D is based principally on insulin administration. The goal is to maintain glycemia within the physiological range, thereby preventing micro- and macrovascular damage that typically occurs in untreated or poorly treated T1D patients. There are different insulin strategies to achieve the goals, intensified therapy and conventional therapy [7]. In addition to its main effect on glucose, insulin also reduces platelet sensitivity to aggregating agents through various mechanisms [8]. Notwithstanding this, T1D patients frequently suffer from arterial thrombosis; therefore, understanding the abnormalities in platelet aggregation in T1D patients is necessary. Although insulin typically reduces platelet aggregation, in the context of insulin resistance or poor glycemic control, its effect may be diminished or even reversed, potentially leading to increased platelet reactivity and a prothrombotic state [9, 10].

In vivo studies have suggested that platelets from diabetic patients exhibit distinct characteristics with abnormal reactivity [11]. The first mentions of increased aggregability in diabetes mellitus date back to 1965 [12]. At the moment, there is no discussion that platelets from diabetic subjects react differently to various agonists of platelet aggregation than those of healthy persons. This abnormal activity seems to be one of the bases for thrombotic events, including acute myocardial infarction and stroke often observed in diabetic patients. Another factor appears to be a lower response of T1D patients to common antiplatelet agents, which may lead to suboptimal therapeutic outcomes [3]. More to that, platelets from diabetic patients can be even truly resistant to clinically used antiplatelet drugs, rendering the therapy more challenging [11]. Therefore, the aims of this study were to (1) describe differences in platelet aggregation in T1D in comparison to healthy controls by using inducers triggering different platelet aggregation pathways, (2) to compare the inhibitory effect of clinically used drugs in the same group, and (3) to see if a promising food flavonoid/polyphenol metabolite 4-methylcatechol (4-MC) [13, 14] can be more active in T1D patients than clinically used acetylsalicylic acid (ASA). Seven very different inducers were selected for monitoring platelet aggregation pathways. Concretely, collagen and ristocetin were chosen as the initial impulses since platelet aggregation starts with the uncovering of subendothelial collagen, and by participation of the von Willebrand factor, whose interaction with its platelet receptor is activated by ristocetin. Three inducers were used to launch more specific cascades, namely adenosine-5-diphosphate (ADP) as it binds to its Gi-type receptors, platelet-activating factor (PAF) as it targets Gq receptors, and thrombin receptor agonist peptide-6 (TRAP). TRAP is a protease-activated receptor 1 (PAR-1) hexapeptide sequence that activates solely the mentioned PAR-1 receptor for thrombin, and does not possess thrombin enzymatic activity. PAR1 is coupled both with G_13_ and Gq cascades [15]. The penultimate inducer selected, arachidonic acid (AA), stimulates an intracellular cascade that leads to the formation of thromboxane A_2_, another potent platelet aggregation inducer that was frequently observed to be increased in diabetic persons [16]. Therefore, a stable agonist of thromboxane receptors, U-46619, was included as the last aggregation trigger in our study. Three clinically used drugs were investigated: the first-line drug ASA blocking the production of thromboxane A_2_ by inhibiting cyclooxygenase 1 enzyme, ticagrelor as a representative directly acting ADP-receptor antagonist, and vorapaxar as a novel drug antagonizing PAR-1 receptors. Experimental compound 4-MC behaves partly similarly to ASA since it also interferes with the formation of thromboxane A_2,_ but the mechanism is different and seems to be based on interference with cyclooxygenase 1 and thromboxane-synthase coupling [17, 18].

## Participants, material, and methods

### Study participants

**Fig. 1:**
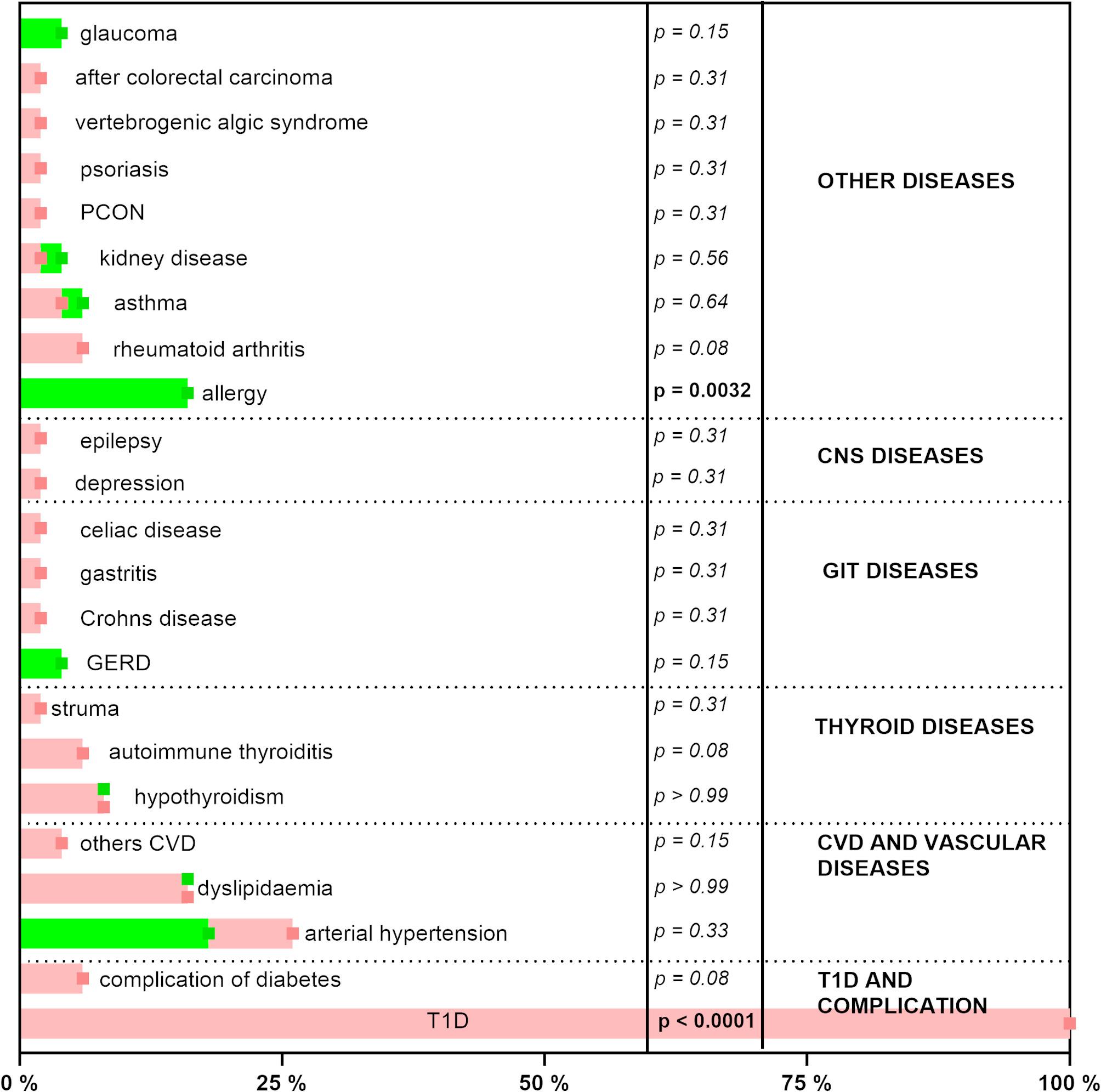
Presence of diseases in type 1 diabetes mellitus patients and generally healthy controls. Healthy donors are marked in green, while diabetic donors are in red. *P*-values for both pictures were calculated by the Chi-square test, with statistical significance in bold. CNS, central nervous system; CVD, cardiovascular disease; T1D, type 1 diabetes mellitus; GERD, gastroesophageal reflux disease; GIT, gastrointestinal tract; PCON, polycystic ovary syndrome

**Fig. 2:**
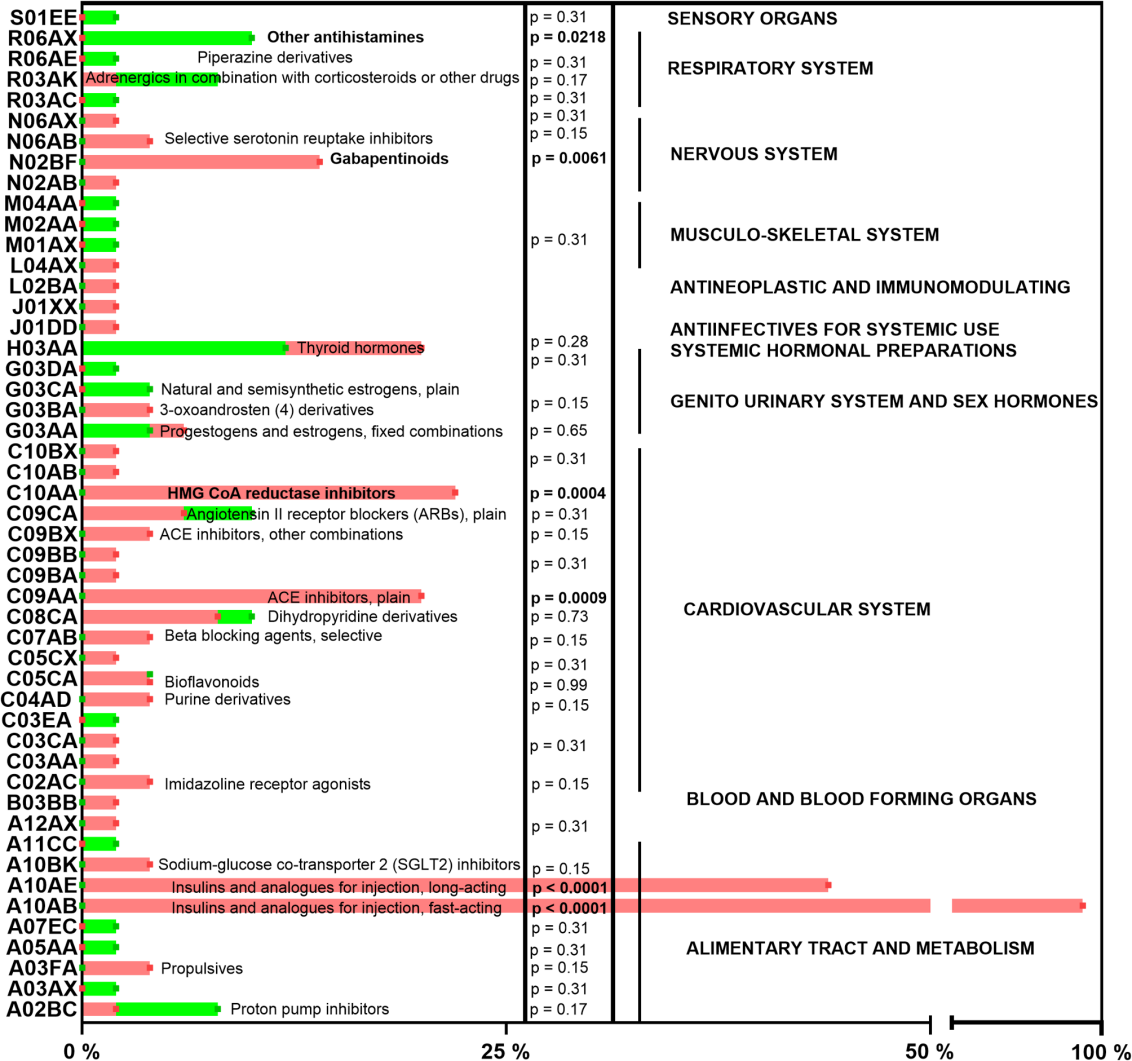
Chronic medication in type 1 diabetes mellitus patients and generally healthy controls. All medications in the investigated groups and drugs were divided according to the appropriate ATC code to the groups by using the ATC/DDD index 2023 from the WHO. Healthy donors are marked in green, while diabetic donors in red. *P*-values for both pictures were calculated by the Chi-square test, with statistical significance in bold

All 50 patients enrolled were suffering from T1D. The diagnosis was based on the value of fasting plasma glucose (≥ 7 mmol/l), positive autoantibodies (anti-GAD 65, ICA2, ICA), and typical clinical symptoms (polyuria, polydipsia, fatigue). The inclusion criteria included, in addition to a diagnosis of T1D, also the age ≥ 18 years. Moreover, these patients could not have severe and acute illnesses (chronic inflammatory diseases, acute infections, and autoimmune or malignant disorders), have been administered any drug known to impact platelet function, and have been treated with non-steroidal anti-inflammatory drugs in the last 14 days (exclusion criteria). Detailed characterization of included donors is shown in Table 1; Figs. 1 and 2. The control group (*n* = 50) was composed of the volunteers from our previous recruitment [18, 19], where the inclusion criteria for this group were age ≥ 18 years, absence of significant illnesses (mild disorders such as allergy, well-treated hypothyreosis, and hypertension were allowed), and subjectively reported good health condition. The exclusion criteria included the presence of any cardiovascular disease (except for the mentioned hypertension), diabetes, metabolic syndrome, and the use of drugs that affect platelet function [18, 19]. All participants signed an informed consent in line with the approval of the ethics committee of the University Hospital in Hradec Králové (No. 202007 S01P from 18 June 2020), and all experiments were ensured according to the latest Declaration of Helsinki.

**Table 1:** Characterization of the control group and type I diabetes mellitus patients (T1D)

		T1D (*n* = 50)	healthy donors (*n* = 50)	*p*-value T1D versus healthy donors
Age	19–74	41 (38–46)	44 (40–47)	*p* = 0.55
Female/male (%)		26/24 (52%/ 48%)	24/26 (48%/ 52%)	*p* = 0.55
BMI	18.5–30+	27.11 ± 4.33	26.92 ± 3.95	*p* = 0.82
Height (cm)	154–195	176.0 (170.0-176.2)	175.0 (172.9-177.5)	*p* = 0.43
Weight (kg)	48–135	81.50 ± 16.50	82.74 ± 14.07	*p* = 0.68
Smokers-n (%)	Yes	11 (22%)	12 (24%)	*p* = 0.81
Biochemical parameters	Serum glucose (mmol/L)	6.84 (6.54–8.31)	5.05 (4.99–5.30)	***p*** **< 0.0001**
	LDL-C (mmol/L)	2.77 ± 0.92	3.45 ± 0.69	***p*** **< 0.0001**
	TC (mmol/L)	4.68 (4.51–5.14)	5.42 (5.27–5.76)	***p*** **< 0.0001**
	HDL-C (mmol/L)	1.47 ± 0.45	1.51 ± 0.36	*p* = 0.62
	TG (mmol/L)	0.96 (1.10–1.76)	1.37 ± 0.88	*p* = 0.15
	Creatinine in serum (µmol/L)	76.99 (73.97–96.52)	76.03 (74.44–83.22)	*p* = 0.97
	Creatinine in urine (mmol/L)	10.72 ± 6.35	10.65 ± 4.77	*p* = 0.97
	HbA1c (mmol/mol)	62.44 ± 18.74	not measured	–

Significant differences are indicated in bold. BMI, body mass index; HbA1c, glycated haemoglobin; HDL-C, serum HDL cholesterol; LDL-C, serum LDL cholesterol; n, number of patients; TC, total serum cholesterol; TG, serum triglycerides. Body mass index was calculated with the known formula: weight [kg]/(height [m])^2^. *P*-values were calculated by parametric or non-parametric tests based on the distribution of values; for smoking the Chi-square test was used; statistical significance is emphasized in bold. Data are shown as mean ± SD or median (interquartile range) related to Gaussian or non-Gaussian distribution. Percent values are related to the total number of subjects

### Blood and urine collection

Blood draws were performed from fasting (≥ 6 h) donors in the morning (at 7–8:30 a.m.). Alcohol was not allowed 24 h before the blood draw. Blood samples for aggregation experiments were collected into tubes containing heparin sodium (17 IU/mL), while blood samples for biochemical analyses were obtained in tubes containing a clotting accelerator. A small volume of the first-morning urine sample was collected from all donors as well.

### Chemicals

#### Agonist of platelet aggregation

Platelet-activating factor-16 (PAF), ristomycin monosulfate (ristocetin), and 9,11-dideoxy-11α,9α-epoxymethanoprostaglandin F_2α_ (U-46619) were purchased from Sigma-Aldrich (St. Louis, MO, USA). ADP, AA, and TRAP were bought from Roche (Basel, Switzerland). Collagen was obtained from Diagnostica, a.s. (Prague, Czechia).

#### Inhibitors of platelet aggregation

ASA, ticagrelor, and 4-MC were purchased from Sigma-Aldrich, while vorapaxar was from Selleck Chemicals GmbH (Planegg, Germany). All antiplatelet drugs were dissolved in dimethyl sulphoxide (DMSO) to keep the same conditions.

#### Solvents and other chemicals

DMSO was bought from Penta (Prague, Czechia), ultrapure water (Mili-Q RG) was prepared by using Merck Millipore (Massachusetts, USA), 0.9% sodium chloride (saline) was purchased from B. Braun (Melsungen, Germany), whereas D-glucose was obtained from Penta (Prague, Czechia) and D-mannitol from Sigma-Aldrich.

### Aggregation experiments

All experiments were performed in whole human blood using the impedance aggregometer Multiplate^®^ (Roche, Switzerland). Heparinized whole blood was used for aggregation experiments. Experiments were always started precisely 30 min after the blood draw. A total of 300 µL of blood sample was mixed with an equal volume of pre-warmed 0.9% sodium chloride solution. This mixture was incubated with a clinically used antiplatelet drug (ASA, vorapaxar, or ticagrelor), 4-MC, or the solvent DMSO for 3 min at 37 °C. Platelet aggregation was triggered using one of seven different inductors with various mechanisms of action (Table S1). The reaction was monitored for 6 min. The aggregation response was quantified by AUC (area under the curve).

### Measurement of biochemical parameters

Glucose, total cholesterol, HDL cholesterol, LDL cholesterol, and triglycerides were measured in serum using standard commercial enzymatic kits employing the Cobas 8000 system (Roche, Basel, Switzerland). Creatinine was determined in both serum and urine. Analysis was carried out using the Prominence LC 20 HPLC set with the SPD-M20A Shimadzu diode array detector (Shimadzu, Kyoto, Japan). As the stationary phase, two monolithic columns RP-18e (4.6 mm × 50 mm, 3.0 mm × 100 mm) were connected together in combination with a 15 mM phosphate buffer as the mobile phase. Creatinine was detected at 235 nm using diode array detection [20].

### Mechanistic experiments mimicking hyperglycaemia and hyperosmolarity in healthy donor samples

For further analysis, blood samples from a total of 14 healthy volunteers (22–34 years old, 36% male) were obtained. Glucose level was measured by a glucometer (Wellion LUNA TRIO Marz, Austria). Experiments were performed similarly to aggregation experiments described above with the exception of the addition of glucose solution in saline instead of pure saline. Glucose was incubated with blood for 3 min at 37 °C before initiation of platelet aggregation by pipetting an inducer. Additional experiments were performed with mannitol; the principle was the same as with glucose reported above, solely mannitol was added instead of glucose to see the possible impact of hyperosmolarity.

### Statistical analysis

GraphPad Prism 10.0.2. (San Diego, California, USA) was used for all data analyses. To confirm or reject the Gaussian distribution, either the Shapiro-Wilk test or the Kolmogorov-Smirnov test was employed according to the number of samples. Subsequently, parametric sample t-test or unpaired sample t-test, and the Wilcoxon matched-pairs test or the Mann-Whitney test were used in comparison of two dependent or independent samples, while one factorial ANOVA or the Kruskal-Wallis test was employed in comparison of more than two independent samples. Chi-square tests were used in categorical analyses. Correlations were analysed using the Pearson correlation test, and a simple linear regression was performed in the case of a significant difference. In case of hyperglycemia and mannitol experiments, two-way ANOVA followed the Šídák’s multiple comparisons test was employed for concentration-dependent comparison of both treatments.

## Results

### Group comparison

T1D patients and the generally healthy control group were well-balanced in basic characteristics. There were no significant differences between them in terms of age, BMI, gender, proportion of smokers, and serum levels of HDL cholesterol and triglycerides (Table 1). T1D patients had logically higher fasting serum glucose levels. Contrarily, generally healthy controls had significantly higher serum levels of total and LDL cholesterol (Table 1). None of the generally healthy volunteers had a previous diagnosis of hypercholesterolemia, but 8 out of 50 volunteers had a higher value than 6.2 mmol/L of total cholesterol in their blood samples. Controls had a significantly higher prevalence of allergy and corresponding use of antihistamines (Figs. 1 and 2). Concerning medication, T1D patients were logically treated with insulins: 47 patients were treated with fast-acting insulins (Anatomical Therapeutic Chemical code /ATC/ A10AB), and 22 of them with long-acting insulins (A10AE) using different commercially available insulin pumps. Solely 2 out of 50 T1D patients were also administered other antidiabetic drugs, namely sodium-glucose co-transporter 2 inhibitors (A10BK). There were also other logical differences in general medication. More diabetic persons were treated with angiotensin-converting enzyme inhibitors (C09AA), gabapentinoids (N02BF: pregabalin and gabapentin), and HMG CoA reductase inhibitors (C10AA: statins) (Fig. 2).

### The effect of common antiplatelet therapy and comparison with 4-MC in T1D

**Fig. 3:**
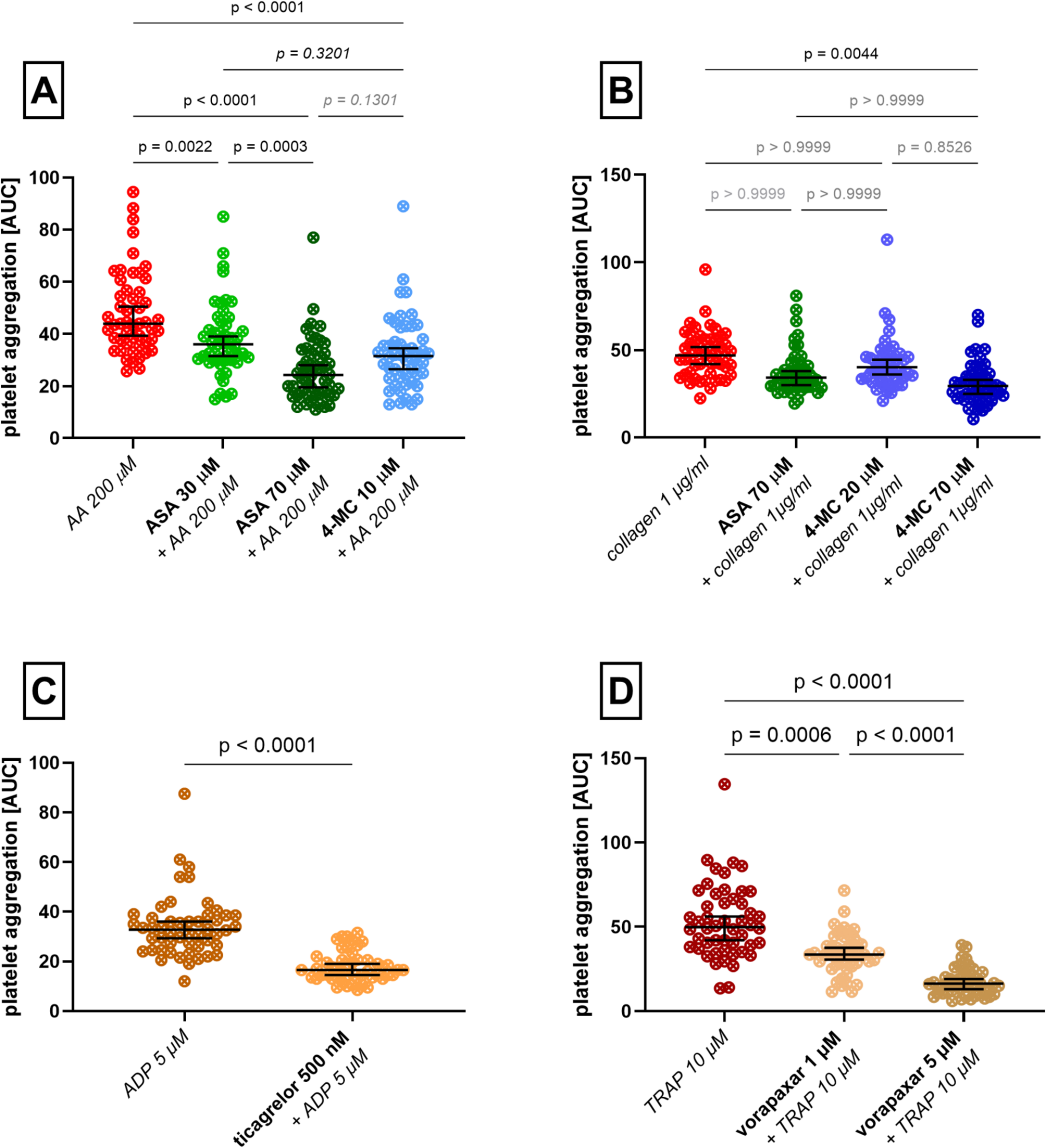
Platelet aggregation and responses to antiplatelet compounds in type 1 diabetes mellitus patients. **A** aggregation induced by arachidonic acid (AA) after pre-treatment with DMSO, acetylsalicylic acid (ASA), or 4-methylcatechol (4-MC). **B** aggregation induced by collagen after pre-treatment with DMSO, ASA, or 4-MC. **C** aggregation induced by ADP after pre-treatment with DMSO or ticagrelor. **D** aggregation induced by TRAP after pre-treatment with DMSO or vorapaxar. AUC, area under the curve; PAF, platelet-activating factor-16; TRAP, thrombin receptor agonist peptide-6; U-46619, 9,11-dideoxy-11α,9α-epoxymethanoprostaglandin F_2α_, *n* = 50. Statistical significance is shown in black, and insignificant differences are in grey. The results are presented as medians with 95% confidence intervals

The standard drug ASA was able to reduce platelet aggregation in our group of T1D, measured as AUC and induced by AA at a clinically achievable concentration of 30 µM by 18.2% [95% confidence interval of the median: 11.4–28.4]. The effect of the flavonoid metabolite 4-MC at a concentration of 10 µM was numerically better than that of ASA. It caused a reduction of 28.4% [21.6–39.8]. Only when the concentration of ASA was elevated to 70 µM, i.e., a concentration not achievable in systemic circulation from low-dose ASA tablets [21, 22], the effect of ASA, accounting for a reduction of 44.9% [36.4–55.7] prevailed numerically but not significantly over 4-MC (Fig. 3A).

A similar situation was observed with collagen (Fig. 3B). Under these conditions, higher concentrations of both compounds must have been used [19]. However, in contrast to our previous research in healthy controls [18], neither 4-MC in a concentration of 20 µM (n.s. reduction by 14.2% [5.2–23.3]) nor ASA in a concentration of 70 µM (n.s. reduction by 27.0% [19.1–36.1]) were able to decrease significantly platelet aggregation under these conditions. Only 4-MC in a concentration of 70 µM was active as it significantly reduced platelet aggregation by 37.1% [29.7–46.7].

4-MC was also able to block ristocetin-induced platelet aggregation (Supplementary Figure S1). Also, the other two conventional drugs, ticagrelor and vorapaxar, were active (Fig. 3CD). Ticagrelor reduced aggregation by 49.6% [42.0–55.7] while vorapaxar in a lower concentration of 1 µM by 32.7% [24.6–38.7] and in a higher concentration of 5 µM by 67.3% [61.8–73.9], both when compared to the positive controls (solvent DMSO with a corresponding inducer).

### Correlation between biochemical and anthropological parameters with platelet aggregability in T1D patients, and the impact of glycemia

**Fig. 4:**
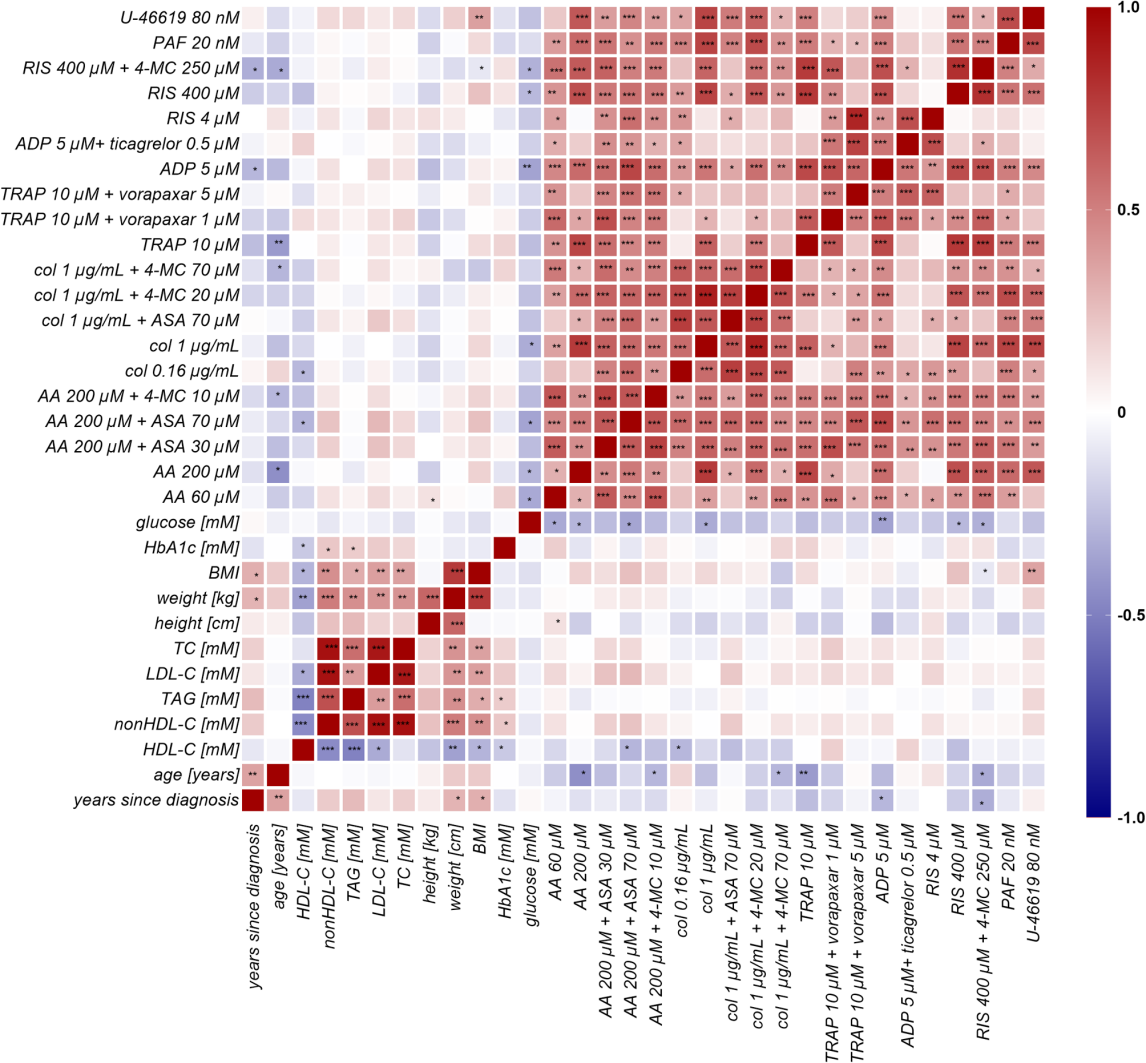
Pairwise correlation scheme. The correlation scheme between platelet aggregation and biochemical/anthropometric parameters. Colour intensity indicates the degree of correlation– Pearson correlation coefficient (r_P_). Positive values are denoted by red colour while negative values by blue colour, and magnitude is depicted as the intensity of colours. AA, arachidonic acid; ADP, adenosine-5-diphosphate; ASA, acetylsalicylic acid; BMI; HDL-C, serum HDL cholesterol; LDL-C, serum LDL cholesterol; nonHDL-C; serum nonHDL cholesterol; PAF, platelet-activating factor-16; TC, total cholesterol; TG, triglycerides; TRAP, thrombin receptor agonist peptide-6; U-46619, 9,11-dideoxy-11α,9α-epoxymethanoprostaglandin F_2α_; 4-MC, 4-methylcatechol, *n* = 50. *P*-values were calculated by the Pearson-correlation test, *p* = 0.05 *, *p* = 0.001 ** and *p* = 0.0001 ***

**Fig. 5:**
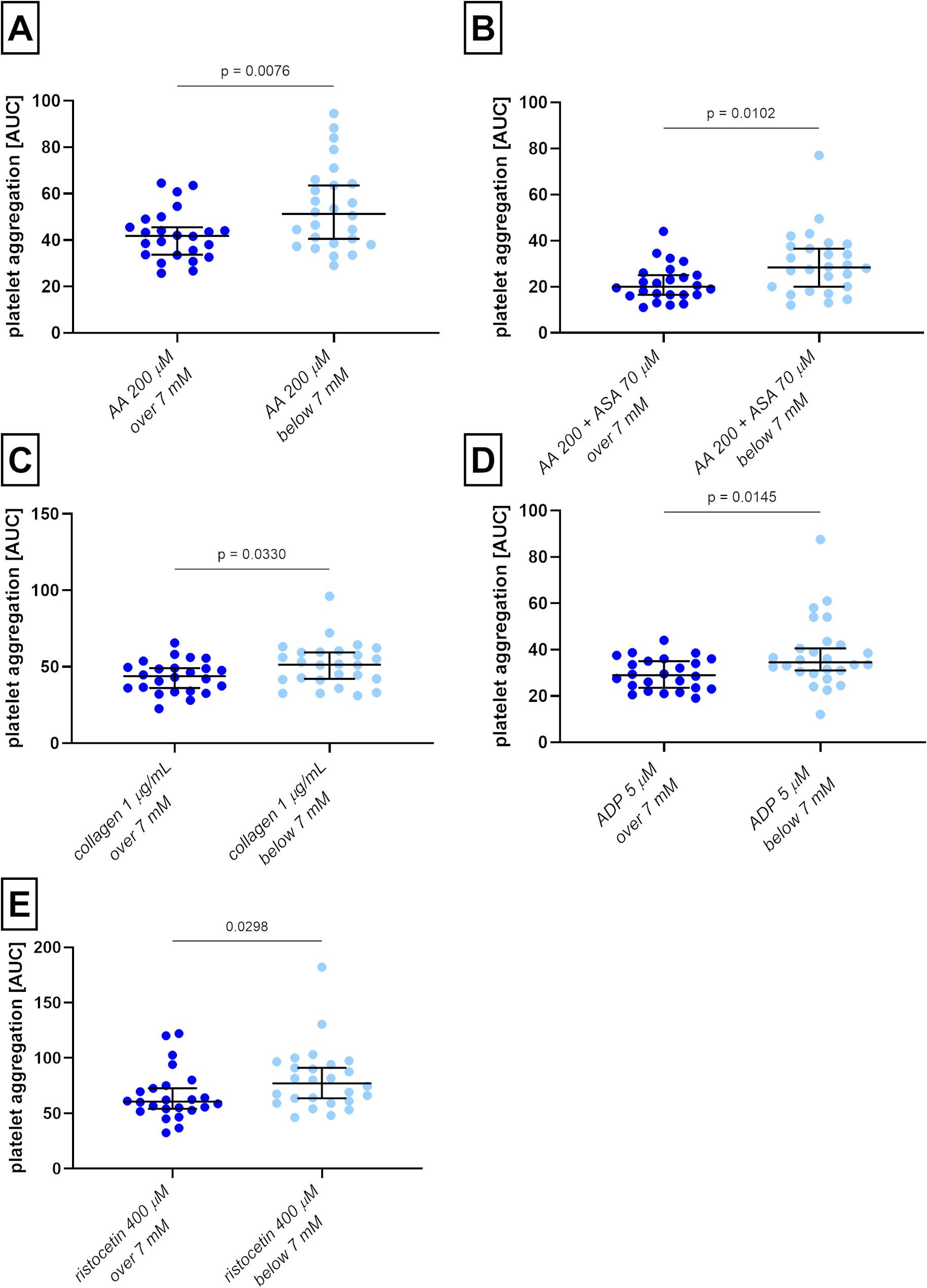
Platelet aggregation in samples from diabetic patients with normal (< 7 mM) and high glucose levels (> 7 mM). **A** aggregation induced by arachidonic acid (AA), **B** aggregation induced by AA after pre-treatment with acetylsalicylic acid (ASA), **C** aggregation induced by collagen, **D** aggregation induced by ADP, **E** aggregation induced by ristocetin. *AUC* area under the curve, *n* = 24 in the group over 7 mM, *n* = 26 in the group bellow 7 mM. The results are presented as medians with 95% confidence intervals

As diabetes affects platelet aggregability, correlations between fasting glucose levels and platelet aggregation were awaited. Several correlations were indeed found (Fig. 4), but they were unexpectedly negative, which means that higher glycemia meant at least acutely lower aggregation (Supplementary Data Figure S2 ABC). This phenomenon was significant for AA, ADP, collagen, and ristocetin. We decided to follow it in more detail, and hence, samples from patients based on a cut-off diabetic glycemia value of 7 mM were divided into 2 groups. 24 out of a total of 50 T1D patients had fasting glucose levels higher than 7 mM at the time of blood draw, and 7 donors reached even more than 10 mM. All four mentioned inductors still induced stronger platelet aggregation in patients with lower glucose levels (Fig. 5). Also, the effect of ASA in a concentration of 70 µM on AA-induced aggregation was weaker in patients with glucose levels below 7 mM, while such a phenomenon was not observed in the case of 4-MC (Fig. 5 and Supplementary Data Figure S3-4). Due to lower aggregation in the T1D group, the effect of ASA was re-assessed in every patient individually by a percent decrease compared to the AA blank. By this analysis, the difference was no longer significant (Supplementary Figure S5).

Similarly, we divided T1D patients according to HbA1c into two groups: over and below 53 mmol/mol; the latter should mean well-treated diabetes. 32 out of 50 T1D patients had HbA1c over 53 mmol/mol, and 2 donors reached more than 130 mmol/mol. There were, however, no differences between those groups of patients with solely one exception of 4-MC at a higher concentration in collagen-triggered reaction (Supplementary Data Figure S6-7). Concerning lipidic parameters, no correlations were found with the exception of 2 negative correlations for HDL-C. Higher HDL-C led to a lower aggregation triggered by the lower dose of collagen and improved the effect of ASA in AA-induced platelet aggregation (Supplementary Data Figure S8). Age correlated well with BMI, while duration of the disease with both BMI, weight, and age. Age had more correlations with platelet aggregation, and all were negative, which means that lower aggregation was observed in higher age (Supplementary Data Figure S9). In order to determine whether there is a relationship between the duration of the disease and platelet aggregation, the T1D patients were divided into two groups with almost the same number of cases, one with a disease duration of less than 23 years and the other with a duration of 23 years or more. There was, however, no difference in any aggregation parameters between these groups (Supplementary Data Figure S10).

**Fig. 6:**
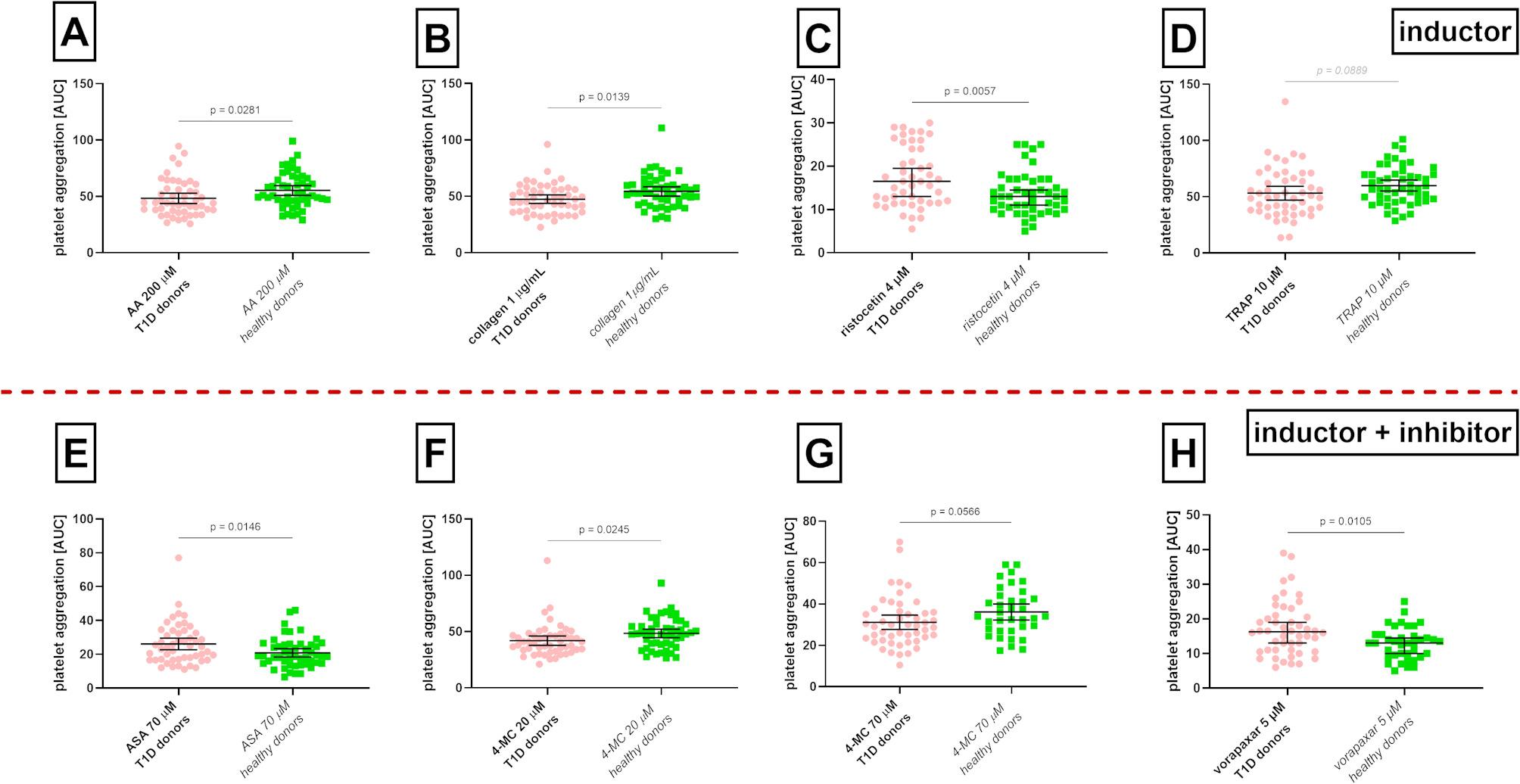
Platelet aggregability between type 1 diabetes mellitus patients (T1D) and age-matched healthy donors. **A** aggregation induced by arachidonic acid (AA), **B** aggregation induced by collagen, **C** aggregation induced by a low dose of ristocetin, **D** aggregation induced by thrombin receptor agonist peptide-6 (TRAP), **E** aggregation induced by arachidonic acid after pre-treatment with acetylsalicylic acid (ASA), **F** and **G** aggregation induced by collagen after pre-treatment with 4-methylcatechol (4-MC), **H** aggregation induced by TRAP in blood samples pre-treated with vorapaxar. AUC, area under the curve; *n* = 50 in both groups. The results are presented as medians with 95% confidence intervals

**Fig. 7:**
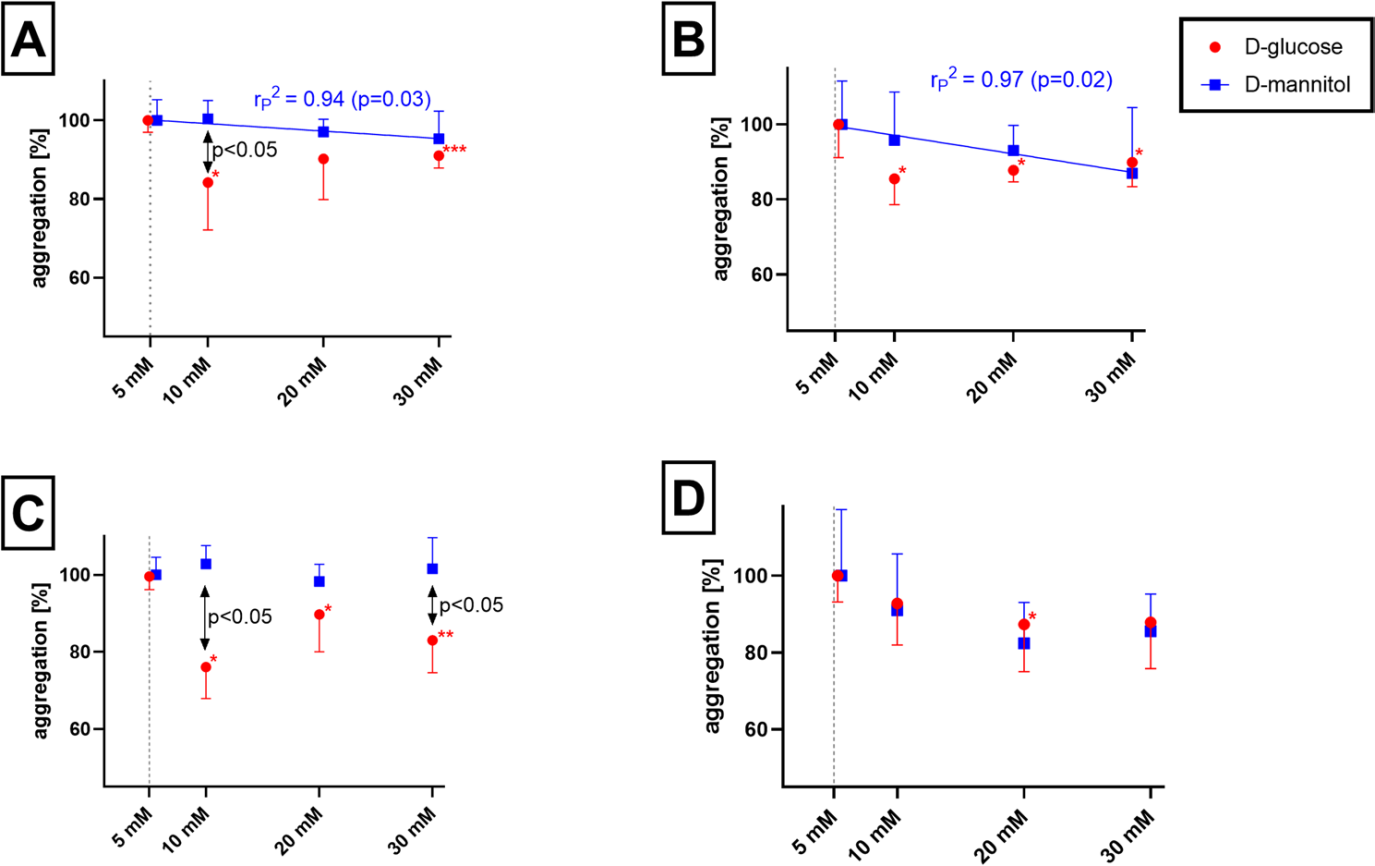
Acute impact of high glucose and mannitol on platelet aggregation. **A** adenosine-5-diphosphate (ADP) 5 µM, **B** U-46619 80 nM, **C** thrombin receptor agonist peptide-6 (TRAP) 10 µM. **D** platelet activating factor (PAF) 20 nM. * *p* < 0.05; ** *p* < 0.01, *** *p* < 0.001 versus the blank. Every agonist was tested in 3 different volunteers. Results are shown as averages ± SD. Equations for a decrease in platelet aggregation (Y) with increasing concentrations of mannitol (X) are as follows: Y = − 0.19*X + 101.1 (ADP) and Y = − 0.49*X + 102.0 (U-46619)

### Platelet aggregability in patients suffering from T1D compared with generally healthy donors

In the next analysis, the results of T1D samples were compared with samples from 50 generally healthy volunteers. Based on our correlation study, it can be inferred that high glucose levels may decrease platelet aggregability. This assumption was confirmed in the case of three agonists (AA, collagen, and TRAP) when platelet aggregability was lower in a group of T1D (Fig. 6 ABD). Only ristocetin showed the opposite result, but this was solely observed in a very low dose, which does not normally induce aggregation (Fig. 6C). Contrarily to inducers, the inhibitory effect of ASA on AA-triggered and vorapaxar on TRAP-induced platelet aggregation in the highest tested concentrations of 70 and 5 µM, respectively, was lower in diabetic persons than in healthy donors, suggesting some resistance of diabetic persons to these drugs (*p* = 0.01, Fig. 6EH). On the other hand, the effect of 4-MC on collagen-induced platelet aggregation was higher at a lower employed concentration of 20 µM (*p* = 0.025) or tended to be higher (70 µM, *p* = 0.057) in diabetic patients than in healthy persons, suggesting its possible advantage in T1D patients (Fig. 6FG). Similar to other conventional drugs, there was a tendency for ticagrelor to have a lower effect in T1D patients (*p* = 0.051). All graphs showing non-significant differences are shown in Supplementary Data Figures S11 and S12. When data were again analyzed as individual changes in every patient, the results were essentially similar, solely there was no difference between healthy and T1D patients in the case of 4-MC and collagen (Supplementary Data Figure S13).

### Possible interference by the presence of hypertension and concomitantly used drugs

To mimic a real population, particularly in higher age categories, our generally healthy control also included 9 volunteers having hypertension, which was managed pharmacologically. Even if there was no significant difference between the prevalence of hypertension in generally healthy donors and T1D patients (18 vs. 26%, Fig. 1), there was a difference in the frequency of use of angiotensin-converting enzyme inhibitors (Fig. 2). As their possible influence on platelets cannot be excluded, subanalyses were performed. We first excluded hypertensive subjects solely from the control group and then from both the healthy and T1D groups. Both analyses brought essentially similar results, which differed solely mildly in p values (Supplementary Data Table 2). There were 3 exceptions: (1) 4-MC in a concentration of 70 µM which tended to be more potent in T1D patients in collagen-induced platelet aggregation in the whole group analysis (*p* = 0.057), became significantly more potent after the exclusion of both hypertensive patients from the generally healthy group (*p* = 0.04) and all hypertensive subjects (*p* = 0.02), (2) The difference in TRAP aggregation between healthy and diabetic persons were insignificant in whole donor analysis (*p* = 0.09), but achieved significance after exclusion of hypertensive subjects from both groups (*p* = 0.047, lower aggregation in T1D group), (3) The effect of ticagrelor on ADP-induced platelet aggregation which was nearly significant when all donors were analysed (*p* = 0.051) and insignificant (*p* = 0.09) when all hypertensive cases were excluded, became significant (*p* = 0.025) when solely “healthy” hypertensive volunteers were excluded.

**Table 2:** Table summarizing the effects of acetylsalicylic acid (ASA) and 4-methylcatechol (4-MC)

	Absolute decrease in healthy	Absolute decrease in T1D	Relative decrease in healthy	Relative decrease in T1D
AA 200 µM + ASA 30 µM	24.02 (9.80–34.31) %	18.18 (11.36–28.41) %	21.77 (14.29–31.63) %	12.68 (6.17–21.05) %
AA 200 µM + ASA 70 µM	63.08 (53.92–65.69) %	44.89 (36.36–55.68) %	66.66 (56.07–69.60) %	43.15 (35.71–50.42) %
AA 200 µM + 4-MC 10 µM	46.08 (35.29–54.90) %	28.41 (21.59–39.77) %	48.00 (36.92–59.51) %	26.45 (17.24–35.52) %
Collagen 1 µg/mL + ASA 70 µM	28.30 (14.15–35.85) %	27.00 (19.01–36.06) %	27.16 (20.18–33.05) %	21.28 (11.11–26.14) %
Collagen 1 µg/mL + 4-MC 20 µM	10.38 (2.83–16.98) %	14.22 (5.16–23.27) %	9.48 (2.70–14.41) %	10.89 (6.13–15.37) %
Collagen 1 µg/mL + 4-MC 70 µM	33.96 (22.64–44.34) %	37.13 (29.67–46.72) %	37.80 (27.03–44.70) %	31.37 (26.26–40.37) %

AA, arachidonic acid; T1D, diabetes mellitus type 1

Absolute decrease means a percent change of medians of the whole group versus the corresponding blank sample (either AA or collagen), while relative decrease the average of individual decreases calculated in every blood donor versus the corresponding blank sample. Results are presented as medians with 95% confidence intervals of the mean. The absolute decrease was calculated as 100%—(median of AUCs of the effects of the selected antiplatelet drug / median of AUCs of the corresponding inducer), while the relative decrease was calculated as median of individual results expressed as 100%—% inhibition (AUC of the effect of the antiplatelet drug/AUC of the inducer)

In the case of exclusions of sartans or ACEi, again, rather minor changes, mostly related to the degree of significance, were observed. Similarly to the exclusion of hypertensive patients, 4-MC (70 µM) became more efficient in T1D patients than in generally healthy controls after stimulation with collagen (*p* = 0.01 vs. *p* = 0.057 in all blood donors) when sartans were excluded. In addition, (1) TRAP was causing milder platelet aggregation in T1D than in controls (*p* = 0.02 vs. *p* = 0.09 in all donors), vorapaxar in a lower concentration was more active in T1D (*p* = 0.035 vs. *p* = 0.15) and again similarly to hypertensive analysis, ticagrelor became more active in the control group but the change was minimal (*p* = 0.0497 vs. *p* = 0.051). Removing all 14 ACEi-treated T1D patients, also did not produce very marked differences. As in the case of hypertensive patients and sartans, 4-MC in a concentration of 70 µM became more potent in T1D patients (*p* = 0.02 vs. *p* = 0.057). In addition, vorapaxar 5 µM and ASA 70 µM (collagen) become equally active in both tested groups (*p* = 0.09 vs. *p* = 0.01 and *p* = 0.07 vs. *p* = 0.015, respectively).

Additionally, diabetic persons were being administered more frequently by gabapentinoids and statins, while generally healthy persons by antihistamines. Removal of all 11 statin-treated patients from the T1D group resulted solely in two changes in significance again, as in the above-mentioned cases: the effect of 4-MC in a concentration of 70 µM on collagen became significant although marginally (*p* = 0.046 vs. *p* = 0.057), and similarly to sartans, vorapaxar (1 µM) was more potent in T1D patients (*p* = 0.045 vs. *p* = 0.15). Exclusion of 7 patients treated with gabapentinoids resulted solely in one change: ristocetin in a higher concentration caused lower aggregation in T1D than in healthy controls (*p* = 0.04 vs. *p* = 0.28). Removal of 6 generally healthy controls using antihistamines led to the same effect with ristocetin (*p* = 0.03 vs. *p* = 0.28) and resulted in a higher effect of 4-MC (70 µM) in the case of collagen in the T1D group (*p* = 0.04 vs. *p* = 0.057), as in most cases of other exclusion analyses.

### Effect of the addition of glucose and mannitol on the aggregation of platelets from healthy patients

In order to see if high glucose concentration itself might be responsible for the above-observed differences in platelet aggregation, blood samples from an additional 11 healthy volunteers were treated shortly with glucose in order to achieve 10, 20, and 30 mM concentrations in blood before administering inducers with or without 4-MC and ASA pretreatment. Four out of seven inducers, ADP, TRAP, U-46,619, and PAF, had lower aggregation responses with higher glucose levels compared to physiological glycemia (Fig. 7, Supplementary Data Figure S14). No clear concentration dependency was observed. To see if solely hyperosmolarity might be responsible, mannitol was used instead of glucose. Interestingly, and in contrast to glucose, mannitol had no effect on TRAP-triggered platelet aggregation while it dose-dependently decreased platelet aggregation induced by ADP and U-46619. Only in the case of PAF, the effect of mannitol copied that of glucose (Fig. 7). The effect of 4-MC was glucose-dependent as it increased with increasing glycemia (*p* = 0.03), in contrast to solely n.s. tendency in the case of ASA (Supplementary Data Figure S15).

## Discussion

T1D is a recognized risk factor for cardiovascular disease (CVD). However, its impact on platelet aggregation is multifaceted and can vary among individuals. Metabolic factors such as hyperglycaemia, hyperlipidaemia, and insulin resistance are associated with increased platelet reactivity. These conditions can lead to a prothrombotic state with an elevated risk of thrombotic events. Conversely, insulin therapy plays a crucial role in modulating platelet function. In well-controlled T1D, insulin can reduce platelet aggregation by enhancing nitric oxide production and improving endothelial function. Both hyper- and hypoaggregation can be encountered in T1D-treated patients due to the variability in individual responses [23–26].

It is known that high glucose level in T1D is a risk factor for developing angiopathy, which leads to atherosclerosis and CVD [27]. Additionally, many studies reported that platelets from diabetic patients are more sensitive to different inducers of platelet aggregation [3, 28, 29]. Our results did not confirm such data, but corroborated the lower response of ASA and vorapaxar, two clinically used antiplatelet drugs, in T1D patients when compared to generally healthy controls. A lower reactivity of platelets in relation to increasing levels of glucose was rather unexpected [3, 28, 29], but on the other hand, it should be emphasized that previous data were almost exclusively based on experiments with platelet-rich plasma or purified platelets. Such experiments can be burdened by the loss of some platelets during the centrifugation procedure. In fact, platelets from diabetic donors are undergoing apoptosis more commonly, and hence, there is a more rapid platelet turnover than in healthy people. This results in an increased proportion of younger platelets in blood, which are more active [30, 31]. It was also documented that a higher proportion of immature platelets and larger platelets are observed in diabetic patients [32]. Logically, the centrifugation procedure can result in the loss of some platelets and give an incomplete platelet aggregation pattern. For this reason, we selected a more advanced impedance testing approach that enables the measurement of platelet aggregation in a more biologically relevant environment, whole blood.

Currently, it is not possible to decipher in detail the process associated with the lower reactivity of platelets in response to high glucose levels. Much previously available data were obtained from animal studies or human clinical trials, where platelets were not solely exposed to higher levels of glucose chronically, but to other factors, like inflammation, which very likely played a role. There is one study showing that the addition of glucose to platelets from healthy donors causes a lower platelet response to thrombin in platelet-rich plasma [33]. We have performed a similar simple experiment to see if other inducers can also be associated with the same phenomenon. Indeed, a mild decrease in platelet aggregation of maximally 20% extent was observed in 4 out of the 7 inducers used, including TRAP (Fig. 7). As this reaction was very rapid, the responsible processes must also be fast. They might include hyperosmolarity, generation of oxidative stress, and/or uncoupling processes. Indeed, (1) hyperosmolarity mediated by different chemical compounds led to a similar drop in thrombin platelet aggregation as glucose, (2) [33] hyperosmolarity blocked rises in intracellular calcium in platelets [34]. Our experiments confirmed the partial impact of osmolarity. There are, however, differences between simple hyperosmolarity, which was induced in our experiments by mannitol, and hyperglycaemia. Interestingly, PAF-triggered platelet aggregation was decreased by both glucose and mannitol to the same extent, but glucose had contrarily to mannitol a platelet aggregation attenuating effect even at a final concentration of 10 mM in the case of the other three inducers. There are no previous data on the mechanisms, possibly with an exception of ADP, which is associated primarily to Gi-receptor. Gi uncoupling was observed in a streptozocin model of T1D [35] and hence, it might take place also in platelets and mediate lower platelet reactivity. An increase in oxidative stress due to hyperglycaemia was observed under different experimental conditions [36, 37], was associated with suppression of endothelium-dependent vasodilation [37], and hence alteration of platelet function can be similarly anticipated. This could be a factor responsible for the difference observed between mannitol and glucose. Other processes, such as changes at the gene transcription level and proinflammation, occur in endothelial cells [38] but are not expected to happen in platelets, given the absence of a nucleus. They can, however, contribute to platelet dysfunction in a whole in vivo model or humans. The need for a more detailed study is also apparent from our results, e.g. the lower response to ADP in hyperglycemia, which can be potentially caused by the above-mentioned Gi receptor uncoupling, was observed in our acute experiments (Fig. 7A) and also in population data (Fig. 5D), but the same phenomenon was seen with AA or collagen solely in population study (Fig. 5AC) pointing out that acute hyperglycemia cannot explain the lower reactivity of platelets in all cases.

Regardless, even if there is an inconsistency between experiments performed with isolated platelets and our whole blood investigation concerning reaction to inducers, there is a very good agreement about resistance to clinically used drugs. In particular, the resistance to ASA is a known phenomenon. A study by Mehta et al. investigated ASA resistance in 92 T1D patients and revealed that 21.7% of these patients exhibited signs of ASA resistance, indicating a diminished platelet response to ASA therapy. A slightly lower percentage of resistance (16.2%) was observed in 111 type 2 diabetes mellitus patients [39]. Similarly, 21.5% of patients were designated as ASA non-responders and 16.9% as semi-responders in a larger study of 172 type 2 diabetes mellitus patients [40]. The resistance phenomenon is not well understood and can be caused by pharmacokinetic as well as pharmacodynamic aspects [41]. Pharmacokinetic aspects, such as lower absorption of ASA in the case of administration of proton pump inhibitors, can be excluded in our study, as we incubated blood samples directly with ASA. There are several theories trying to describe the pharmacodynamic mechanism of ASA resistance [41]; some mechanisms might be common for non-diabetic and diabetic persons. Regardless, diabetic persons seem to have higher levels of thromboxane A_2,_ and hence, its production can escape the blockade of cyclooxygenase 1 by ASA. In fact, thromboxane production in platelets is higher in diabetic patients than in healthy donors [16], and analogously, urine thromboxane excretion was still higher in diabetic patients treated with a low-dose ASA than in healthy controls [42]. As 4-MC affects the same pathway as ASA but targets cooperation between thromboxane synthase and cyclooxygenase 1, it might overcome the ASA resistance phenomenon, as was at least partly observed in our study. Previously, we have observed advantageous antiplatelet properties of 4-MC over ASA in a fertilized hen egg in vivo-like model [17] as well as in healthy donors and a small group of familiar hypercholesterolemia patients [14, 18]. As far as we know, this is the first study showing the antiplatelet effect of a microbial metabolite of human dietary phenolics in diabetic persons. A summary comparison of the effects of ASA and 4-MC is reported in Table 2. Moreover, 4-MC seems to be free of toxicity toward platelets [17], cell cultures up to a concentration of 100 µM [13], and red blood cells (up to 1 mM, unpublished results). 4-MC was also shown to affect multiple but not all platelet aggregation pathways, which is an advantage. In addition to AA, collagen and ristocetin it decreases platelet aggregation induced by PAF, calcimycin, bryostatin 1, thapsigargin and dithiothreitol. On the other hand, it has no effect on platelet aggregation triggered by ADP, TRAP or U-46619 [17, 18].

In addition to differences between ASA and 4-MC, our data showed that ticagrelor tended to have lower activity in T1D patients. Ticagrelor targets the ADP-receptor, which is linked to Gi protein, and in addition to inhibiting of production of cAMP with adenylyl cyclase, also starts the phosphatidylinositol 3-kinases/protein kinase B pathway [43]. This is particularly an important finding, especially in light of resistance issues related to clopidogrel, which were solely partly explainable by known pharmacokinetic aspects [15]. Vorapaxar blocks thrombin-based activation of the PAR-1 receptor, which triggers the Gq- and G_13_-protein pathway. Interestingly, the product of the arachidonic acid-based pathway, thromboxane A_2_ binds to receptors associated with Gq and G_13_ receptors. It is of interest to point out a common pathway associating ASA with vorapaxar. ASA blocks the production of thromboxane A_2_, and vorapaxar targets a similar Gq/G_13_ pathway as thromboxane A_2_. In principle, this pathway might be associated with lower effects of both drugs in DTM1 patients observed in our study.

In terms of possible resistance, the selected concentrations of clinically used drugs should also be discussed. We used concentrations that, based on our previous experiments in a lower number of blood donors, caused around half inhibition of platelet aggregation to observe possible differences in both directions in a larger data set of patients [17, 19]. The concentrations employed were always of clinical relevance. The used concentration of ticagrelor (500 nM) fits into its plasma levels in patients not experiencing bleeding with an interquartile range of 140–342 ng/mL (~ 270–650 nM) [44]. In the case of vorapaxar, the concentration was higher (1 µM) than can be expected in common patients, as a 40 mg single dose resulted in a maximal plasma level of 435 ng/mL (~ 0.88 µM, geometric mean) [45]. This is quite interesting, as both healthy individuals [19] as well as our T1D patients can be resistant even to this high concentration. A concentration of 5 µM was used solely for experimental purposes to study whether a higher concentration can overcome the observed resistance. A similar situation occurred with ASA. The concentration employed in our study was slightly higher than plasma levels observed after repeated doses of ASA (25 µM, 325 mg daily [21]), which again emphasizes the resistance as an important issue. Due to resistance observed in our previous study, we included a higher concentration of 70 µM, which is achievable clinically solely after administration of higher doses of ASA [22].

Our recruited generally healthy donors who had some minor illnesses were logically treated with some drugs. Similarly, T1D patients were administered several cardiovascular and other drugs. It is well known that concomitant pharmacotherapy can potentially influence the outcome. In fact, there is a hypothesis that lipid-lowering drugs, as well as angiotensin-converting enzyme inhibitors, might have positive impacts on platelet aggregation [46]. There are some controversies in terms of statins, which generally do not seem to affect platelet aggregation [47]. Contrarily, gabapentin was clearly shown to act as an antiplatelet drug, but in concentrations that are not commonly achievable in clinical settings, but they can be detected after high doses [48, 49]. To eliminate these possible confounders, patients taking these drugs were excluded from our subanalyses. The results suggested that neither statins nor ACEi inhibitors had a clear antiplatelet effect, as their exclusion resulted solely in the improvement of the activity of some drugs in the diabetic population (Supplementary Table S2). The effect was hence opposite to that which would happen if they had antiplatelet potential. Similarly, the exclusion of gabapentinoids from the T1D group resulted solely in a decrease in the platelet reaction of these patients to ristocetin. This again suggests that no impact of gabapentinoid administration on our antiplatelet outcomes.

We must underline that our study had some limitations. (1) Our generally healthy group was not fully healthy, as it is very difficult or almost impossible to recruit fully healthy persons in higher age categories. (2) We were not able to perform other analyses due to the already relatively high volume of blood needed for our experiments (biochemical, aggregatory, coagulation - the latter data were already published [50]). (3) We have not measured glycated haemoglobin in the control group. (4) The druggability of 4-MC is unclear. The strong antiplatelet properties of 4-MC come from the research trying to explain the cardiovascular effects of a diet rich in polyphenols. Since polyphenols are poorly absorbed in the parent form, their claimed cardiovascular effects result likely from the formation of small phenolics by human microbiota, which are then well absorbed. 4-MC was by far the most potent of these phenolics in terms of antiplatelet potential. There is, however, a huge difference between possible cardiovascular protection in mild-risk persons, which can be mediated by lower concentrations of 4-MC, and its use as an antiplatelet drug in patients already having cardiovascular disease or being at a high risk for developing it. Limited pharmacokinetic data showed that 4-MC reached 3.5 µM concentrations after consumption of cranberry polyphenol-rich juice, but it was detected solely in the sulfate form [51]. Hence, the concentration of 70 µM used in some our experiments is unphysiological. As 4-MC is probably produced in the colon slowly, its absorption can be associated with a rapid and complete conjugation. Another situation might be after oral administration of high 4-MC doses, which need not result in complete conjugation due to the saturation effect. Such a pharmacokinetic study has not yet been performed, but given the risk of a potential failure to achieve concentrations in tens of µM which will be likely needed for antiplatelet effect in diabetic persons, we are also considering its derivatives which [13] might be less prone to rapid conjugation.

## Conclusion

This study showed that blood from T1D patients had a lower reactivity to platelet aggregation inducers but contrarily was more resistant to clinically used antiplatelet drugs ASA and vorapaxar. This phenomenon seems to be linked with a higher level of glycemia based on both results classifying blood samples with glycemia over 7 and below 7 mM, and acute experiments simulating hyperglycaemia in blood from healthy donors. Contrarily, the effect of 4-MC does not appear to be markedly affected by glycemia, and there was a better or equal response to this compound in T1D patients than in healthy controls, emphasizing that this compound or its derivatives might have the potential for becoming antiplatelet drugs.

## Supplementary Information

Below is the link to the electronic supplementary material.

Supplementary Material 1.

## Data Availability

Data are available at ZENODO (DOI: 10.5281/zenodo.15479513).
