# Supplementary material for: Acetylsalicylic acid and vorapaxar are less active, while 4-methylcatechol is more active, in type 1 diabetic patients compared to healthy controls
Source: Cardiovasc Diabetol. 2025 Aug 7;24:323. doi: 10.1186/s12933-025-02891-6 (PMC12329942; doi:10.1186/s12933-025-02891-6)
Supplement: Supplementary file 1 — Supplementary Material 1. [file 12933_2025_2891_MOESM1_ESM.docx]

**Acetylsalicylic acid and vorapaxar are less active, while 4-methylcatechol is more active, in type 1 diabetic patients compared to healthy controls.**

Markéta Paclíková^#1^, [Lukáš Konečný](https://link.springer.com/article/10.1007/s10557-023-07455-y#auth-Luk__-Kone_n_-Aff1)^#2^, [Alejandro Carazo](https://link.springer.com/article/10.1007/s10557-023-07455-y#auth-Alejandro-Carazo-Aff1)^2^, [Kateřina Matoušová](https://link.springer.com/article/10.1007/s10557-023-07455-y#auth-Kate_ina-Matou_ov_-Aff3)^3^, [Lenka Kujovská Krčmová](https://link.springer.com/article/10.1007/s10557-023-07455-y#auth-Lenka_Kujovsk_-Kr_mov_-Aff3-Aff4)^3,4^, [Vladimír Blaha](https://link.springer.com/article/10.1007/s10557-023-07455-y#auth-Vladim_r-Blaha-Aff5)^1^, Alena Šmahelová^1^ and [Přemysl Mladěnka](https://link.springer.com/article/10.1007/s10557-023-07455-y#auth-P_emysl-Mlad_nka-Aff1)^2^

^1^ The 3^rd^ Department of internal medicine-metabolic care and gerontology, University Hospital and Faculty of Medicine in Hradec Králové, Charles University, Hradec Králové, Czech Republic

^2^ The department of pharmacology and toxicology, Faculty of Pharmacy in Hradec Králové, Charles University, Hradec Králové, Czech Republic

^3^ The department of clinical biochemistry and diagnostics, University Hospital Hradec Králové, Hradec Králové, Czech Republic

^4^ The department of analytical chemistry, Faculty of Pharmacy in Hradec Králové, Charles University, Hradec Králové, Czech Republic

## **^#^These authors contributed equally.**

*Correspondence: [mladenkap@faf.cuni.cz](mailto:mladenkap@faf.cuni.cz)

**SUPPLEMENTARY DATA**

**18 pages**

**Table S1.** Summary of inducers and inhibitors (antiplatelet drugs) and their final concentrations.

|  | | **final concentrations** |
| --- | --- | --- |
| inducers | collagen | 0.16 µg/mL; 1 µg/mL |
|  | arachidonic acid (AA) | 60 µM; 200 µM |
|  | ristocetin | 4 µM; 400 µM |
|  | platelet-activating factor-16 (PAF) | 20 nM |
|  | U-46619 | 80 nM |
|  | adenosine-5-diphosphate (ADP) | 5 µM |
|  | thrombin receptor activating protein-6 (TRAP) | 10 µM |
| antiplatelet compounds (inhibitors) | acetylsalicylic acid (ASA) | 30 µM; 70 µM |
|  | ticagrelor | 0.5 µM |
|  | vorapaxar | 1 µM; 5 µM |
|  | 4-methylcatechol (4-MC) | 10 µM; 20 µM; 70 µM |

**Table S2** Impact of the exclusion of hypertensive patients and patients/donors treated with major groups of drugs.

| analyses | type | p-value all | p-value control - AH | p-value T1D and control - AH | p-value without  ACEi | p-value without  statins | p-value  without  gabapentinoids | p-value without antihistamines | p-value without sartans |
| --- | --- | --- | --- | --- | --- | --- | --- | --- | --- |
|  |  |  | **-9 controls** | **-13 T1D and -9 controls** | **-14 T1D** | **-11 T1D** | **-7 T1D** | **-6 controls** | **-3 T1D and -5 controls** |
| AA 60 µM | MW / MW / MW/ MW / MW / MW/ MW / MW | 0.2201 | 0.1041 | 0.1161 | 0.2257 | 0.1189 | 0.1739 | 0.3095 | 0.3161 |
| AA 200 µM | unp / MW / MW/ MW/ MW/ MW/ MW/ MW | **0.0281** | **0.0141** | **0.0158** | **0.0240** | **0.0186** | **0.0142** | **0.0094** | **0.0038** |
| AA 200 µM + ASA 30 µM | unp / MW / unp/ MW/ unp/ MW/ MW/ unp | 0.4160 | 0.4635 | 0.2583 | 0.2901 | 0.6478 | 0.3659 | 0.1395 | 0.2496 |
| AA 200 µM + ASA 70 µM | unp / MW / MW/ MW/ unp/ MW/ MW/ MW | **0.0146** | **0.0111** | **0.0340** | 0.0674 | **0.0119** | **0.0283** | **0.0487** | **0.0292** |
| AA 200 µM + 4-MC 10 µM | MW / MW / MW/ MW/ MW/ MW/ MW/ MW | 0.3773 | 0.1955 | 0.1433 | 0.2921 | 0.0577 | 0.2038 | 0.4958 | 0.3492 |
| Collagen 0.16 µg/mL | MW / MW / MW/ MW/ MW/ MW/ MW/ MW | 0.5375 | 0.7297 | 0.2331 | 0.2429 | 0.2736 | 0.4957 | 0.3960 | 0.4728 |
| Collagen 1 µg/mL | unp / MW / MW/ MW/ MW/ MW/ MW/ MW | **0.0139** | **0.0355** | **0.0196** | **0.0081** | **0.0083** | **0.0110** | **0.0082** | **0.0092** |
| Collagen 1 µg/mL + ASA 70 µM | unp / MW / MW/ MW/ unp/ MW/ MW/ MW | 0.1257 | 0.2663 | 0.2100 | 0.0629 | 0.1544 | 0.1970 | 0.1598 | 0.1754 |
| Collagen 1 µg/mL + 4-MC 20 µM | unp / MW / MW/ MW/ unp/ MW/ MW/ unp | **0.0245** | **0.0113** | **0.0044** | **0.0030** | **0.0056** | **0.0059** | **0.0014** | **0.0047** |
| Collagen 1 µg/mL + 4-MC 70 µM | unp / MW / MW/ MW/ MW/ MW/ MW/ MW | 0.0566 | **0.0397** | **0.0239** | **0.0235** | **0.0464** | 0.0738 | **0.0395** | **0.0134** |
| ADP 5 µM | unp / MW / MW/ MW/ unp/ MW/ MW/ unp | 0.4960 | 0.3281 | 0.2561 | 0.5130 | 0.5446 | 0.4350 | 0.2577 | 0.3309 |
| ADP 5 µM + ticagrelor 500 nM | MW / MW / MW/ MW/ MW/ MW/ MW/ MW | 0.0508 | **0.0253** | 0.0928 | 0.1387 | 0.1064 | 0.0632 | 0.1256 | **0.0497** |
| Ristocetin 4 µM | MW / MW / MW/ MW/ MW/ MW/ MW/ MW | **0.0057** | **0.0027** | **0.0040** | **0.0287** | **0.0113** | **0.0097** | **0.0135** | **0.0080** |
| Ristocetin 400 µM | unp / MW / unp/ MW/ unp/ MW/ MW/ MW | 0.2788 | 0.0783 | 0.0598 | 0.1957 | 0.0913 | **0.0437** | **0.0295** | 0.0527 |
| Ristocetin 400 µM + 4-MC 240 µM | MW / MW / unp/ MW/ unp/ MW/ MW/ MW | 0.9340 | 0.9005 | 0.5146 | 0.3057 | 0.3474 | 0.6246 | 0.8767 | 0.7466 |
| TRAP 10 µM | unp / MW / unp/ MW/ unp/ MW/ unp/ unp | 0.0889 | 0.0517 | **0.0470** | 0.1265 | 0.1140 | 0.0565 | 0.0505 | **0.0242** |
| TRAP 10 µM + vorapaxar 1 µM | MW / unp / unp/ unp/ unp/ unp/ unp/ unp | 0.1534 | 0.1840 | 0.1301 | 0.1175 | **0.0452** | 0.0626 | 0.0742 | **0.0354** |
| TRAP 10 µM + vorapaxar 5 µM | MW / MW / MW/ MW/ MW/ MW/ MW/ MW | **0.0105** | **0.0060** | **0.0348** | 0.0889 | **0.0498** | **0.0249** | **0.0242** | **0.0468** |
| PAF 20 nM | unp / MW / unp/ MW/ MW/ MW/ MW/ MW | 0.1659 | 0.2929 | 0.1678 | 0.1302 | 0.1157 | 0.1950 | 0.1129 | 0.1232 |
| U-46619 80 nM | unp / MW / MW/ MW/ MW/ MW/ MW/ MW | 0.1514 | 0.1601 | 0.1987 | 0.2165 | 0.3152 | 0.2066 | 0.2514 | 0.2223 |

AH – arterial hypertension, MW – Mann-Whitney, T1D – type 1 diabetes mellitus, unp – unpaired T-test

All results had been analysed first using the Shapiro-Wilk test and then with the corresponding test mentioned above.

Green colour – control group showed lower aggregation, red colour – T1D group showed lower aggregation.

**Supplementary Figure S1. Platelet aggregation after ristocetin and response to 4-methylcatechol in type 1 diabetic patients.** Aggregation was induced by ristocetin (400 µM) after pre-treatment with the solvent DMSO or 4-MC. AUC: area under the curve, *n = 50*. The results are presented as medians with 95% confidence intervals.


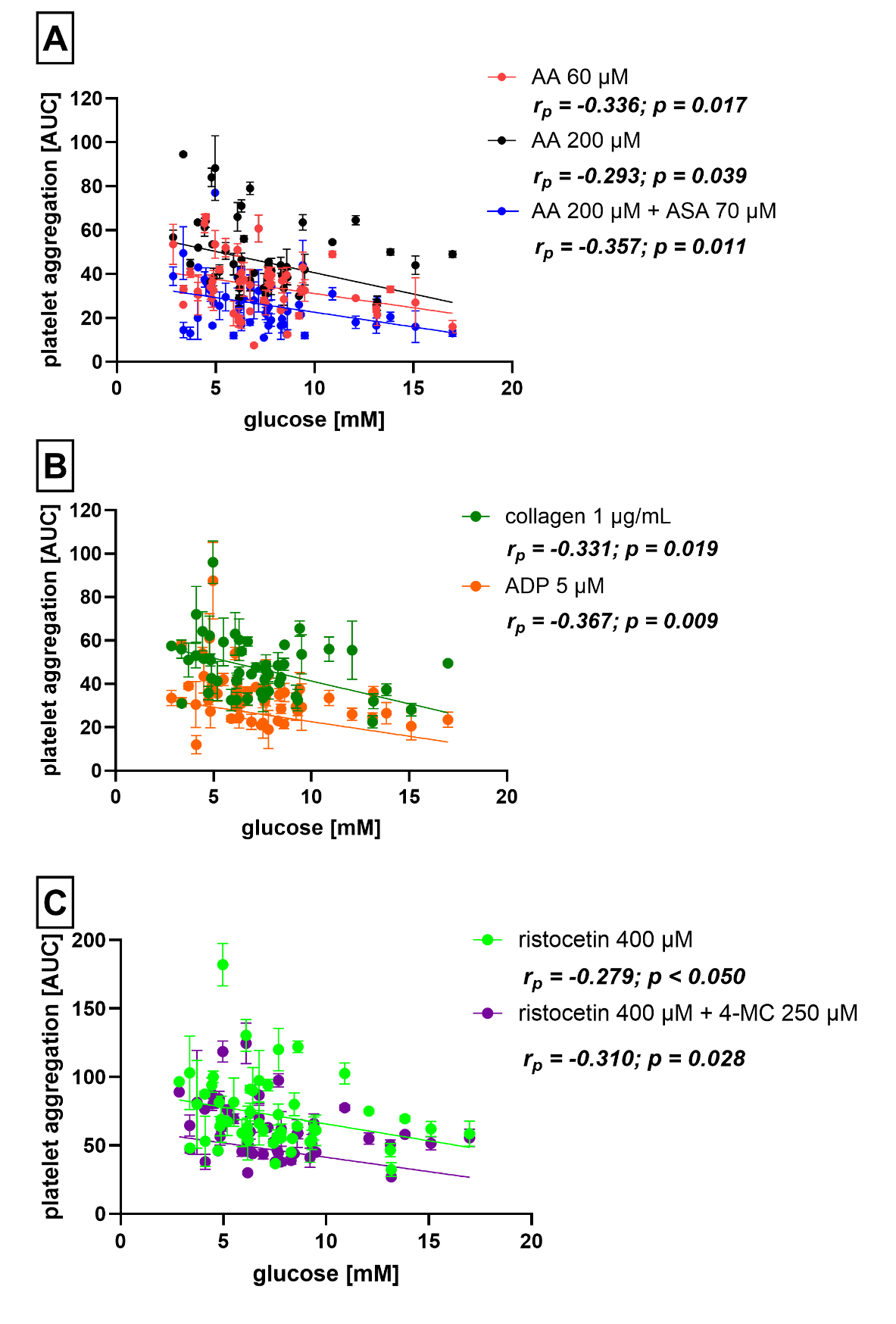


**Supplementary Figure S2. Linear relationships between increasing serum levels of glucose and decreasing platelet aggregability in type 1 diabetes mellitus patients.** AA: arachidonic acid; ADP: adenosine-5-diphosphate; ASA: acetylsalicylic acid; 4-MC: 4-methylcatechol, n = 50

**Supplementary Figure S3. Comparison of platelet aggregation between samples with normal (<7 mM) and high glucose level (>7 mM) – different inducers.**

Aggregation induced by various agonists**.** AA: arachidonic acid; PAF: platelet-activating factor-16; TRAP: thrombin receptor agonist peptide-6; U-46619: 9,11-dideoxy-11α,9α-epoxymethanoprostaglandin F_2α_; AUC: area under the curve, *n = 24* in the group over 7 mM, *n = 26* in the group below 7 mM. The results are presented as medians with 95% confidence intervals.

**Supplementary Figure S4. Comparison of platelet aggregation between samples with normal (<7 mM) and high glucose level (>7 mM) – effect of tested inhibitors of platelet aggregation.**

Aggregation induced by various agonists after pre-treatment with acetylsalicylic acid (ASA), 4-methylcatechol (4-MC), or ticagrelor. AA: arachidonic acid; ADP: adenosine-5-diphosphate; AUC: area under the curve; *n = 24* in the group over 7 mM, *n = 26* in the group below 7 mM. The results are presented as medians with 95% confidence intervals.

**Supplementary Figure S5. Relative changes in arachidonic acid (AA)-platelet induced aggregation after pretreatment with acetylsalicylic acid (ASA) in samples from type 1 diabetic patients with normal (<7 mM) and high glucose levels (>7 mM).** Aggregation was induced by AA (200 µM) after pre-treatment with ASA, *n = 24* in the group over 7 mM, *n = 26* in the group below 7 mM. The results are presented as medians with 95% confidence intervals.


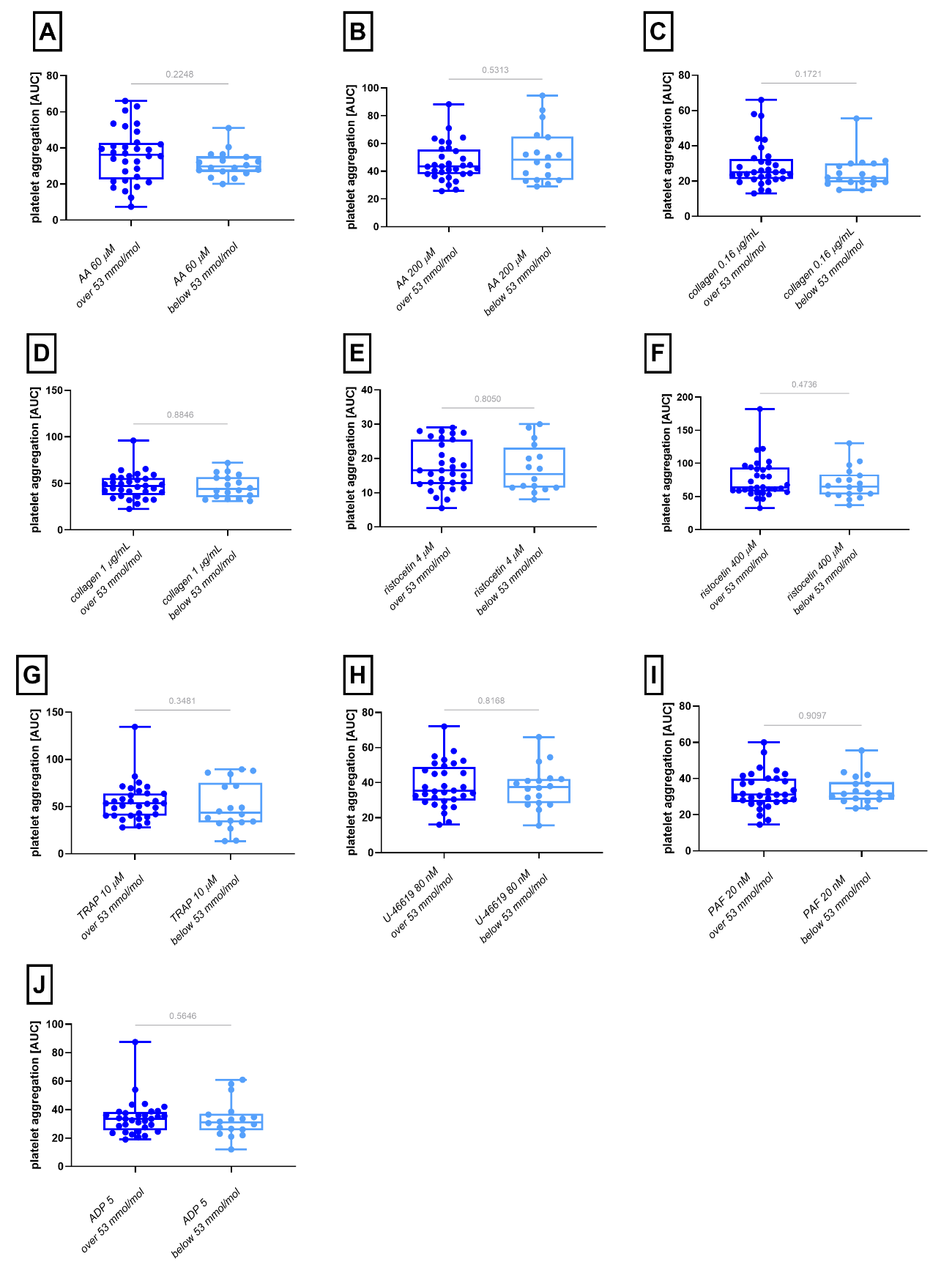


**Supplementary Figure S6. Comparison of platelet aggregation between samples with normal (<53 mmol/mol) and high HbA1c level (>53 mmol/mol) – agonists of platelet aggregation.**

Aggregation induced by various agonists**.** AA: arachidonic acid; PAF: platelet-activating factor-16; TRAP: thrombin receptor agonist peptide-6; U-46619: 9,11-dideoxy-11α,9α-epoxymethanoprostaglandin F_2α_; AUC: area under the curve*, n = 32* in the group over 53 mmol/mol, *n = 18* in the group below 53 mmol/mol. The results are presented as medians with 95% confidence intervals.


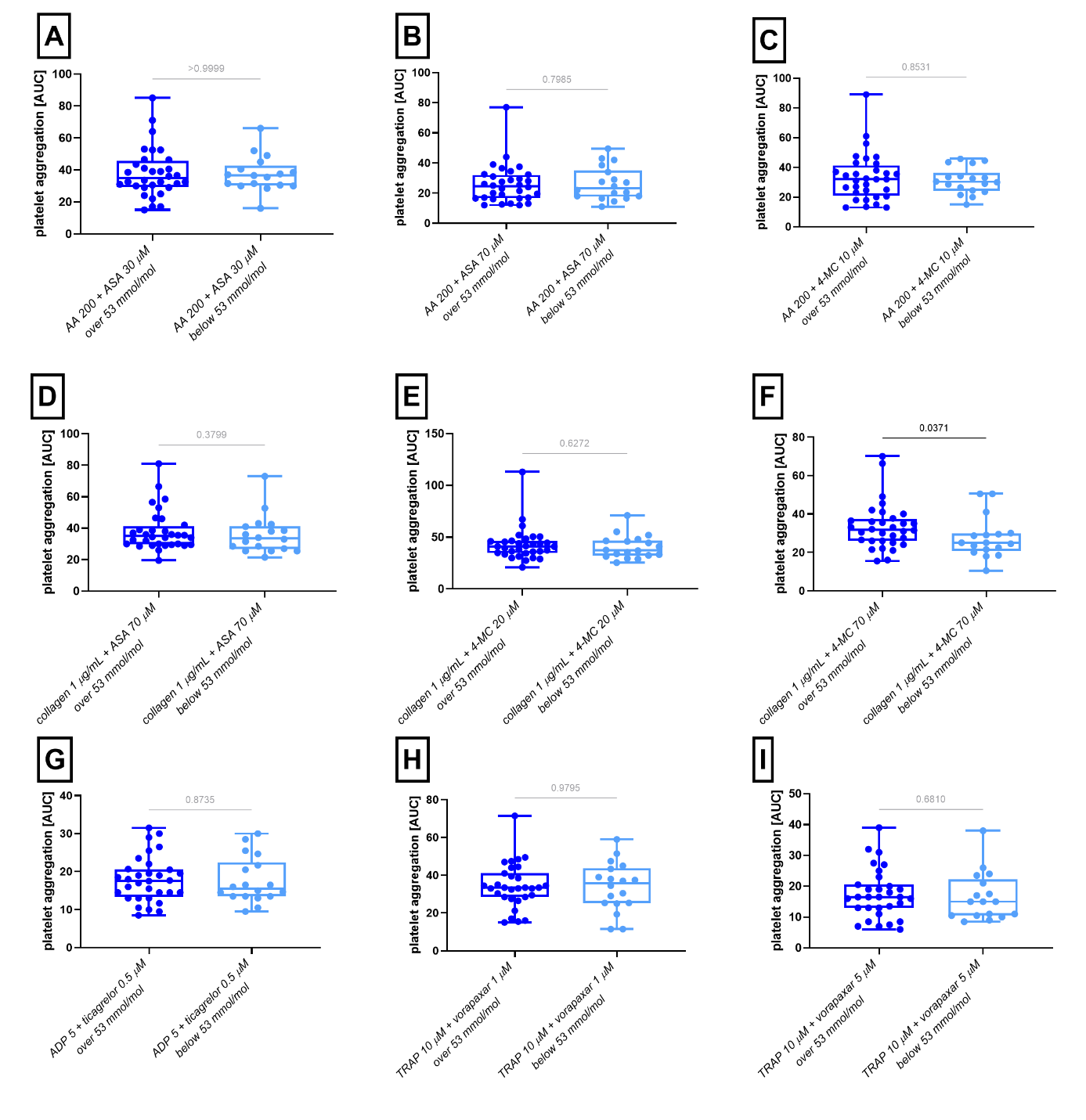


**Supplementary Figure S7. Comparison of platelet aggregation between samples with normal (<53 mmol/mol) and high HbA1c level (>53 mmol/mol) – the effect of antiplatelet compounds.**

Aggregation induced by various agonists after pre-treatment with acetylsalicylic acid (ASA), 4-methylcatechol (4-MC), ticagrelor, and vorapaxar. AA: arachidonic acid; ADP: adenosine-5-diphosphate; AUC: area under the curve; *n = 32* in the group over 53 mmol/mol, *n = 18* in the group below 53 mmol/mol. The results are presented as medians with 95% confidence intervals.

**Supplementary Figure S8. Linear relationships between increasing levels of HDL cholesterol (HDL-C) and decreasing platelet aggregability in type 1 diabetes mellitus patients.** AA: arachidonic acid; ASA: acetylsalicylic acid, n = 50.


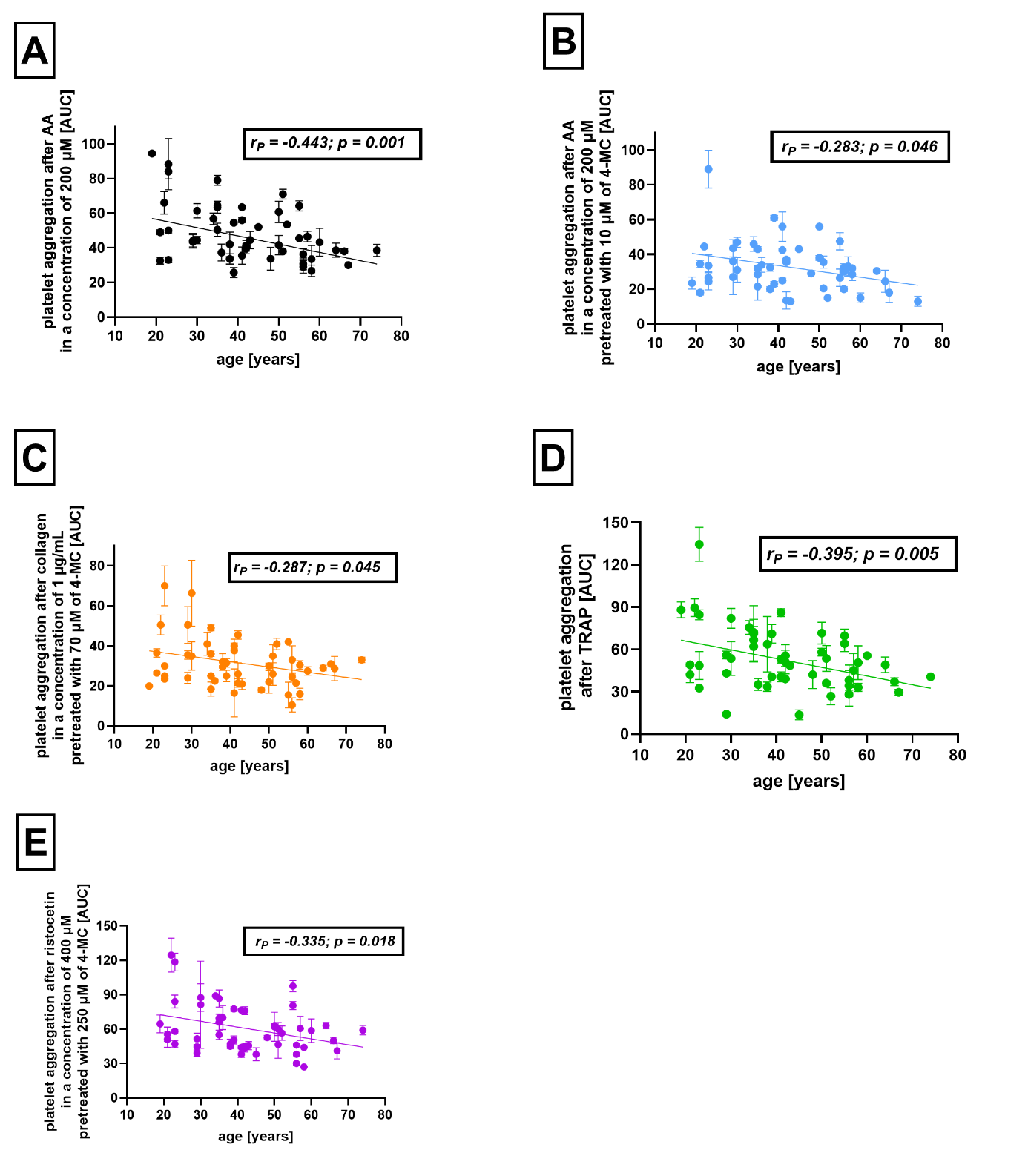


**Supplementary Figure S9. Significant relationships between age and platelet aggregation in type 1 diabetes mellitus patients.** AA: arachidonic acid; 4-MC: 4-methylcatechol, TRAP: thrombin receptor agonist peptide-6; *n = 50*


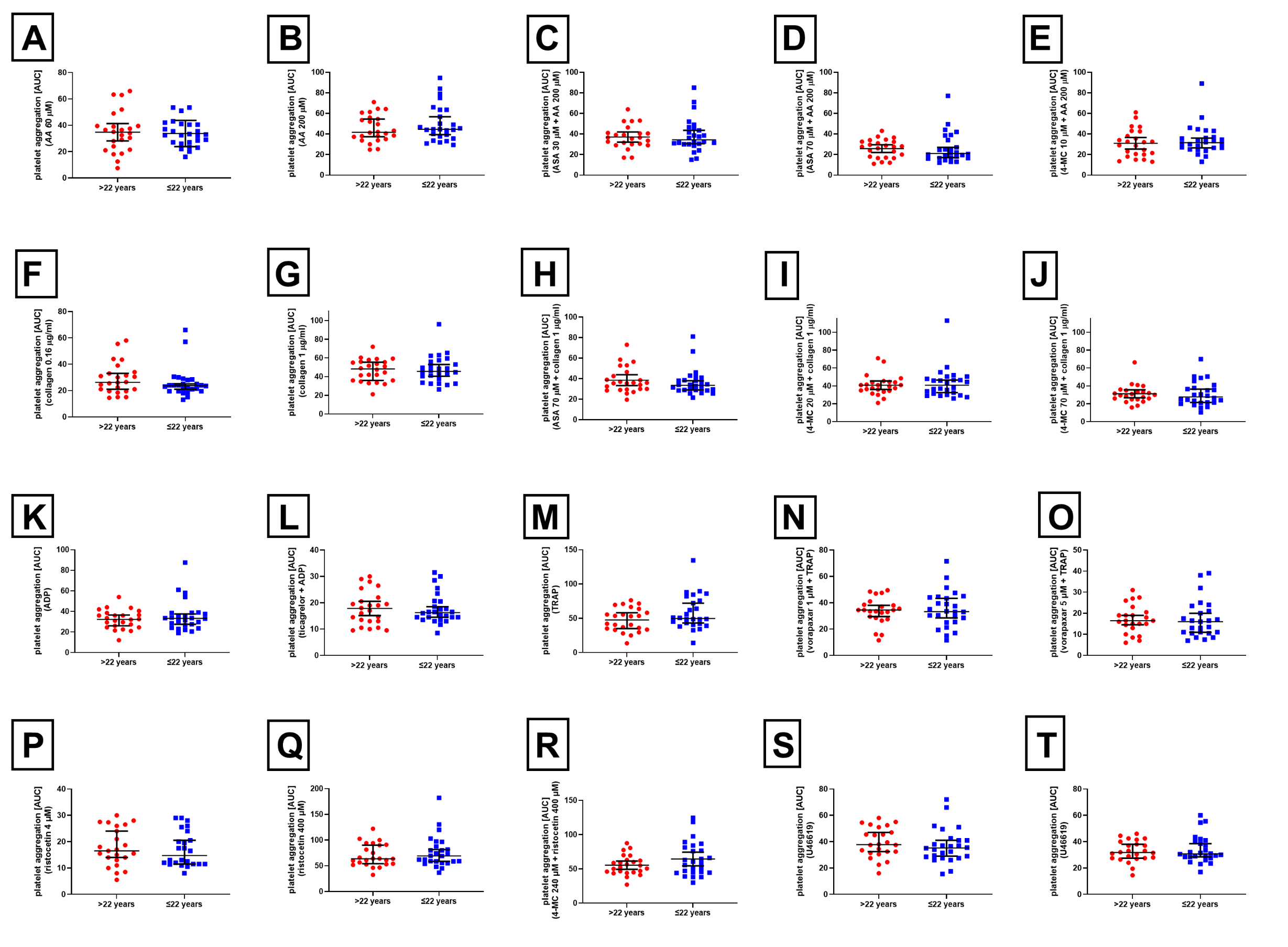


**Figure S10. No effect of type 1 diabetes mellitus duration on platelet aggregation and its inhibition by antiplatelet compounds.** 24 patients had a disease duration of 23 years or more, while 26 had the disease for less than 23 years. Data are shown either as median or mean with 95% confidence intervals based on the result of the Shapiro-Wilk normality test. Based on the normality, the unpaired Student t-test or the Mann-Whitney test was employed for testing the differences. In any case, significant difference was found.

**Supplementary Figure S11. Platelet aggregability between diabetes mellitus patients (T1D) and age-matched healthy donors – insignificant cases.** **A**: aggregation induced by arachidonic acid (AA), **B**: aggregation induced by collagen, **C**: aggregation induced by ristocetin, **D**: aggregation induced by ADP, **E**: aggregation induced by platelet-activating factor-16 (PAF), **F**: aggregation induced by U-46619 (9,11-dideoxy-11α,9α-epoxymethanoprostaglandin F_2α_). ADP: adenosine-5-diphosphate; AUC: area under the curve; *n = 50* in both groups. The results are presented as medians with 95% confidence intervals.

**Supplementary Figure S12. Platelet response to antiplatelet compounds between type 1 diabetes mellitus patients (T1D) and age-matched healthy donors.** **A**: aggregation induced by arachidonic acid after pre-treatment with acetylsalicylic acid (ASA), **B**: aggregation induced by arachidonic acid after pre-treatment with 4-methylcatechol (4-MC), **C**: aggregation induced by collagen after pre-treatment with ASA, **D**: aggregation induced by ristocetin in blood samples pre-treated with 4-MC, **E**: aggregation induced by ADP in blood samples pre-treated with ticagrelor, **F**: aggregation induced by TRAP (thrombin receptor agonist peptide-6) in blood samples pre-treated with vorapaxar. ADP: adenosine-5-diphosphate; AUC: area under the curve; *n = 50* in both groups. The results are presented as medians with 95% confidence intervals.

**Supplementary Figure S13. Relative individual changes in platelet aggregation** **between type 1 diabetes mellitus patients (T1D) and age-matched healthy donors.** **A**: aggregation induced by arachidonic acid after pre-treatment with acetylsalicylic acid (ASA), **B** and **C**: aggregation induced by collagen after pre-treatment with 4-methylcatechol (4-MC), **D**: aggregation induced by TRAP in blood samples pre-treated with vorapaxar. *n = 50* in both groups. The results are presented as medians with 95% confidence intervals. These data represent changes in platelet aggregation calculated in every patient in per cent vs. the corresponding blank.

**Supplementary Figure S14. Impact of high glucose on platelet aggregation.** **A**: arachidonic acid (AA) 200 µM, **B**: collagen 1 µg/mL, **C**: ristocetin 250 µM. *n = 3* different volunteers. Results are shown as averages ± SD.

**Supplementary Figure S15. The impact of high glucose on arachidonic acid-induced platelet aggregation treated with acetylsalicylic acid 70 µM (ASA) and 4-MC (10 µM).** *n=3* different volunteers. Results are shown as average ± SD.
